# Fungal keratitis in patients with corneal ulcer attending Minilik II Memorial Hospital, Addis Ababa, Ethiopia

**DOI:** 10.1186/s12886-016-0330-1

**Published:** 2016-08-30

**Authors:** Tihtina Kibret, Adane Bitew

**Affiliations:** 1 Department of Medical Laboratory Sciences, Tirunesh Beijing Hospital, Addis Ababa, Ethiopia; 2 Department of Medical Laboratory Sciences, College of Health Sciences, Addis Ababa University, Addis Ababa, Ethiopia

**Keywords:** Fungal keratitis, Risk factors, Filamentous fungi, Yeasts

## Abstract

**Background:**

Fungal keratitis is an important cause of corneal blindness all over the world. Although there are several reports on fungal keratitis from developing and developed countries, fungal keratitis in Ethiopia is poorly known. The aim of this study was to determine the prevalence of fungal keratitis and spectrum of fungi implicated in causing the infection.

**Methods:**

The present study was a single institutional cross-sectional study carried out in Minilik II Memorial Hospital eye clinic, Addis Ababa, Ethiopia from September 2014 to August 2015. Corneal scraping was obtained under aseptic condition with sterile 21 gauge needle by an ophthalmologist from patients suspected of microbial keratitis. Each scraping was inoculated onto Sabouraud Dextrose Agar in C-shaped streaks and incubated at 25 °C aerobically for four weeks. Cultures of mycelia fungi were identified by examining macroscopic and microscopic characteristics of their colonies. Yeasts were identified by employing biochemical and assimilation test procedures and using CHROMagar *Candida* culture. All data were coded, double entered and analyzed using SPSS version 20.

**Result:**

Out of 153 cases of microbial keratitis, fungi were recovered from 69 patients giving fungal keratitis prevalence of 45.1. Patients from rural areas were significantly affected than patients in urban regions (*P* = 0.005). Age groups of 25–34 (*P* = 0.017) and 15–24 years (*P* = 0.008) were significantly affected. Fungal keratitis was significantly associated with farmers (*P* = 0.0001), daily laborers (*P* = 0.0001), unemployed (*P* = 0001) and students (*P* = 0.004). Fungal keratitis was statistically associated with trauma (*P* = 0.006), and diabetes (*P* = 0.024). Seventy six fungal isolates were recovered, of which molds accounted 63 (82.9 %) of the total isolates. *Fusarium* and *Aspergillus* species were the two predominant molds accounting 27.6 and 25 % of the total isolates respectively. Yeast isolates accounted only 17.1 %.

**Conclusion:**

High prevalence of fungal keratitis recorded in the present study, highlights the need for nationwide study on fungal keratitis and precise identification of the causative fungi and institution of appropriate treatment strategy.

## Background

Microbial keratitis is an opportunistic, serious sight threatening ocular disease that can be caused by different types of microorganisms [1]. Fungi are one of the most common infective organisms responsible for this morbidity [2]. Currently, fungal keratitis has been recognized as a major global public health problem particularly in developing nations located in tropical and subtropical regions [3] representing up to 6–50 % of all cases of culture proven infectious keratitis [4]. Development and widespread use of broad-spectrum antibiotics and steroids [4], trauma to the eye [5, 6], frequent and prolonged use of contact lens [6], seasonal variation [7], ocular surface disease [6] and underlying diseases that compromise the immune mechanism of the host [8] have been identified as major factors that contribute to the increasing number of fungal keratitis.

Many species of fungi belonging to at least 70 genera have been identified as etiological agents of fungal keratitis [9] in which tropical isolates are most frequently filamentous fungi such as *Aspergillus sp* or *Fusarium sp* accounting 70 % of cases [10]. The diversity of fungi isolated from fungal keratitis, however, varies with geographical regions studied. Filamentous organisms are associated with infections following trauma with vegetable- contaminated matter in tropical and subtropical regions [5, 11] while yeast, especially *Candida sp* predominate in temperate regions [12–14].

High rainfall, longer rainy season and high humidity throughout the year have been identified as favorable environmental conditions for fungal growth in countries located in tropical and sub-tropical regions such as Ethiopia. Moreover, 85 % of the population of the country is engaged in agriculture including the labor-intensive tea and coffee plantation industries. These make, people more vulnerable for corneal ulceration and fungal infection. In spite of these conditions, there is no a single systemic study conducted on fungal keratitis in Ethiopia. Lack of routine fungal culture and experts in the field in health institutions are incriminated as the major factors. To this effect, conducting research to understand the actual magnitude of fungal keratitis and its etiological agents from patients referred all over the country to the only referral eye clinic in the country appears to be timely and appropriate. Thus, this study was designed to determine the magnitude of fungal keratitis and the spectrum of fungi implicated in causing the ulceration. Findings from this study will provide up-to date information on fungal keratitis for evidence-based action aimed at reducing the morbidity of the infection.

## Methods

### Study site and period

The present study was a single institutional cross-sectional study carried out in Minilik II Memorial Hospital eye clinic, Addis Ababa, Ethiopia from September 2014 to August 2015. It is the only referral clinic in the country for the management of infectious keratitis. Willingness to participate in the study, presumptive diagnosis of infectious keratitis and no history of antifungal therapy within 2 weeks prior to their attendance were the inclusion criteria.

### Clinical examination and laboratory investigation

The requisition form was a standard proforma filled up by the ophthalmologist, documenting socio-demographic information, previous treatment, predisposing ocular conditions and related risk factors. Patients enrolled during a routine clinic visit were examined by slit lamp and corneal scraping was obtained under aseptic conditions with a sterile 21 gauge needle, following the instillation of a local anesthetic (tetracaine hydrochloride 0.5 %) by an ophthalmologist at duty. Material obtained from scraping was inoculated directly onto Sabouraud Dextrose Agar (SDA) plates supplemented with chloramphenicol (Oxoid, Basingstoke, UK) in C-shaped streaks under safety cabinet level II. All inoculated plates were then transported to the Department of Medical Laboratory Sciences, College of Health Science, Addis Ababa University and incubated at 25 °C aerobically for four weeks. Culture plates were examined twice a week for any fungal growth.

### Identification

Fungi were identified by studying their microscopic, macroscopic characteristics and by using an array of biochemical and assimilation tests according to Kern and Blevins [15]. Briefly, cultures of mycelia fungi (molds) were identified by examining macroscopic and microscopic characteristics of their colony. Texture, rate of growth, topography and pigmentation of the front and the reverse side of the culture were employed for macroscopic identification. Microscopic identification of mold isolates was performed by placing pieces of a colony from SDA to clean microscopic slide and staining with lactophenol cotton blue. After placing a cover slip, the characteristics of conidia and mycelia of each isolate were studied microscopically. Yeasts were identified by employing an array of biochemical and assimilation test procedures and using CHROMagar *Candida* culture medium (Becton Dickinson) as per the instruction of the manufacturer.

### Statistical analysis

All data from the investigation were coded, double entered and analyzed using SPSS version 20. Descriptive statistics and logistical regressions were used to estimate crude ratio with 95 % confidence interval to the different variables. *P*-value < 0.05 was considered significant.

### Ethical consideration

All ethical considerations and obligations were duly addressed and the study was conducted after the approval of the Department Research and Ethical Review Committee (DRERC) of the Department of Medical Laboratory Sciences, College of Health Sciences, Addis Ababa University and the Addis Ababa City Health Bureau. Informed written consent was obtained from participants before data collection. The respondents were given the right to refuse to take part in the study as well as to withdraw at any time during the study period. All the information obtained from the study subjects were coded to maintain confidentially. When the participants were found to be positive for fungal pathogen, they were informed by the hospital clinician and received proper treatment. Assent form was completed and signed by family member and/or adult guardian for participants under the age of 16 years.

## Results

One hundred fifty three patients suspected of microbial keratitis were examined for fungal keratitis. Out of 153 cases of microbial keratitis investigated, fungi were recovered from 69 patients giving fungal keratitis prevalence of 45.1 %. Demographic characteristics of study subjects and association of fungal keratitis with sex, age, residence, occupation and risk factors are depicted in Table 1. Of 69 patients with fungal keratits, 29 (42.0 %) were female and 40 (58.0 %) were male patients. As shown in Table 1, the association of fungal keratitis with gender was not statistically significant [(OR = 1.139, 95 % CI, 0.599–2.167) (*P =* 0.691]. Forty seven (68.1 %) and 22 (31.9 %) patients with fungal keratitis were inhabitants of rural and urban areas respectively. Residents of rural areas were significantly affected by fungal keratitis than urban residents [(OR = 0.387, 95 % CI, 0.199–0.751) (*P =* 0.005]. Fungal keratitis was the highest (47.8 %) in patients of age group 25–34 followed by age groups of 15–24 (31.9 %). Age groups of 25–34 [(OR = 3.548, 95 % CI, 1.260–9.996) (*P =* 0.017] and age groups of 15–24 [(OR = 4.583, 95 % CI, 1.500–14.001) (*P =* 0.008] were significantly affected with fungal keratitis. Similarly, fungal keratitis was higher in farmers accounting 43.5 %, followed by daily laborers (18.8 %), unemployed (jobless) (17.4 %) students (11.6 %) and surveyor (8.7 %) respectively. The infection was significantly associated with farmers [(OR = 90, 95 % CI, 20.985–385.987) (*P =* 0.0001], daily laborers [(OR = 11.700, 95 % CI, 3.597–38.054) (*P =* 0.0001], unemployed [(OR = 21.600, 95 % CI, 5.647–82.620) (*P =* 0.0001] and students [(OR = 6.000, 95 % CI, 1.755–20.517) (*P =* 0.004]. The mycosis was also significantly associated with trauma [(OR = 6.321, 95 % CI, 1.684–23.751) (*P =* 0.006], and diabetes %) [(OR = 10.833, 95 % CI, 1.374–85.440) (*P =* 0.024].

**Table 1:** Demographic characteristic of study subjects and association of fungal keratitis with sex, age, residence, occupation and risk factors

Demographics	Variables	Number	Fungal keratitis		*P*.value	COR	95 % CI
			Yes	No			
Sex	Female	67	29	38		1	
	Male	86	40	46	0.691	1.139	0.599–2.167
Total		153	69	84			
Age in years	1–14	26	6	20	1		
	15–24	38	22	16	0.008	4.583	1.500–14.001
	25–34	64	33	31	0.017	3.584	1.260–9.996
	35–44	20	8	12	0.221	2.222	0.619–7.974
	>45	5	-	5	NA		
Total		153	69	84			
Residence	Rural	85	47	38	0.005	0.387	0.199–0.751
	Urban	68	22	46	1		
Total		153	69	84			
Occupation	Agriculture	33	30	3	0.0001	90.000	20.985–385.987
	Daily laborer	23	13	10	0.0001	11.700	3.597–38.054
	Unemployed	17	12	5	0.0001	21.600	5.647–82.620
	Student	20	8	12	0.004	6.000	1.755–20.517
	Merchants	7	-	7	-	-	-
	Carpenter	7	-	7	-	-	-
	Cleaner	9	-	9	-	-	-
	Driver	7	-	7	-	-	-
	Secretary	6	-	6	-	-	-
	Teacher	15	-	15	-	-	-
	Surveyor	9	6	3	1		
Total		153	69	84			
Risk factors	Trauma	91	54	37	0.006	6.324	1.684–23.751
	Unidentified	26	1	25	0.146	0.173	0.016–1.836
	Diabetes	7	5	2	0.024	10.883	1.374–85.440
	Contact lens	4	2	2	0.118	8.667	0.577–130.111
	Topical steroids	10	4	6	0.243	2.889	0.486–17.170
	Post-operative	15	3	12	1		
Total		153	69	84			

*COR* crude odds ratio, *Cl* confidence interval

The predisposing risk factors for fungal keratitis and the proportion of yeast and filamentous fungi associated with each risk factor are depicted in Table 2. Ocular trauma was the most common risk factor representing a larger percentage (78.3 %). Sixty one fungi (80.3 %) were isolated from trauma of which 68.4 % were filamentous fungi while 11.9 % were yeast isolates. This was followed by systemic (diabetes) consisting of 6.6 %.

**Table 2:** Distribution of fungal isolates across predisposing risk factors (*n* = 76)

Predisposing factor	Frequency (*n*, %)	Fungal isolates (*n* = 76)		Total (*n*, %)
		Molds (*n*, %)	Yeasts (*n*, %)	
Trauma	54 (78.3)	52 (68.4)	9 (11.9)	61 (80.3)
Systemic (diabetes)	5 (7.2)	4 (5.3)	1 (1.3)	5 (6.6)
Topical steroids	4 (5.8)	3 (4.0)	1 (1.3)	4 (5.3)
Contact lens	2 (2.9)	2 (2.6)	-	2 (2.6)
Post-operative	3 (4.4)	2 (2.6)	1 (1.3)	3 (3.9)
Unidentified	1 (1.4)	-	1 (1.3)	1 (1.3)
Total	69 (100)	63 (82.9 %)	13 (17.1 %)	76 (100)

A total 76 fungal isolates belonging to 13 genera were recovered, of which 13 were mixed cultures. Of the total isolates, mycelia fungi were the most common isolates accounting 82.9 % of the total isolates. *Fusarium* and *Aspergillus* species were the two predominant mycelia fungi consisting of 27.6 and 25.0 % of the total isolates respectively. *Penicillium sp*, *Cladosporium sp* and *Scedosporium* sp accounted 7.9 %, 6.6 and 5.3 %, respectively. Yeast isolates accounted only 17.1 % of the total fungal isolates (Table 3).

**Table 3:** Spectrum of fungal isolates from patients with Fungal keratitis (*n* = 69)

Fungal isolates	Pure culture	Mixed culture	Total isolates	% of the total
*C. albicans*	9	3	12	15.8
*Rhodotorula sp*	-	1	1	1.3
*Acremonium sp*	3	-	3	3.9
*Alternaria sp*	1	-	1	1.3
*A.flavus*	4		4	5.3
*A. fumigates*	3	-	3	3.9
*A. niger*	9	2	11	14.5
*A.terreus*	1	-	1	1.3
*Cladosporium sp*	5	-	5	6.6
*Curvularia spp*.	1		1	1.3
*Fusarium oxysporium*	5	1	6	7.9
*Fusarium solani*	11	2	13	17.1
*Fusarium sp*	2		2	2.6
*Penicillium spp*.	6	-	6	7.9
*Paecilomyces sp*	0	1	1	1.3
*Rhizopus spp*.	1	-	1	1.3
*Scedosporium sp*	1	3	4	5.3
*Sepedonium sp*	1	-	1	1.3
Total	63	13	76	100

## Discussion

Although fungal keratitis accounts for about 50 % of all cases of culture-proven microbial keratins [4], the magnitude of the problem and its etiologic agents are poorly known in Ethiopia. As the result, fungal infections of the cornea have remained challenging for diagnosis and treatment. Thus, this study was carried out with the aim of determining the magnitude of fungal keratitis and the spectrum of the etiologic agents.

Out of 153 patients with infectious keratitis attending Minilik II Memorial Hospital over 12 month period, the prevalence of fungal keratitis was found out to be 45.1 %. Though the prevalence of fungal keratitis in our study was within the reported range, a comparatively high prevalence rate was achieved. Prevalence of fungal keratitis of 30.4 %, 37.5 and 36.8 % were reported in similar studies conducted by Garg et al. [16], Shokohi et al. [17] and Narsani et al. [18] respectively. Even quite a lower prevalence rate of fungal keratitis was reported in a similar study conducted at University Hospital of Taiwan in 2004. Of 476 patients included in the study with microbial keratits, only 13.5 % patients were found out to be infected with fungi [19]. In contrast to our study, Mirshahi et al. [20] and Javadi et al. [21] reported quite higher prevalence rates (83 %) of fungal keratitis. Variation in the prevalence rate of fungal keratitis in our study with earlier studies could have resulted from regional difference of fungal keratitis attributed to culture, geographical location and/or seasonal variations.

The result of this study showed the highest frequency of fungal keratitis (47.8 %) in the age groups of 25 to 34 years that was more or less similar to the observations made by Upadahay and Murthy [22] in Nepal as well as in others studies [23, 24]. Our finding with regard to age was also comparable to the findings reported in many developing countries such as South India [25], North China [26] and Southeast Brazil [27]. This could be explained by the fact that patients in the age group of 25–34 in this study are the main force of manual works, especially in agricultural and outdoor activities.

Our study revealed that 68.1 % of fungal isolates were recovered from rural population. Furthermore, patients engaged in farming accounted 43.5 % fungal isolates followed by daily laborer (18.8 %) together accounting 62.3 % of the total fungal isolates. This is obvious because the rural parts of Ethiopia are inhabited by farmers and daily laborer. Higher incidence of fungal keratitis has also been reported among farmers in South India [22, 25]. A significant association of fungal keratitis with students and unemployed patients is not clear. However, participation of students in farming and tea and coffee plantations during semester breaks and/or every day after class is a common practice in the rural regions of Ethiopia and this could be a possible explanation for a significant association of the infection with students. On the other hand, chronic nature of fungal keratitis could be a possible factor for a significant association of fungal keratitis with unemployed (jobless) patients registered in this study as they might have acquired the infection before they lost their jobs.

Corneal trauma with vegetable contaminated matter has always been identified as a major cause of fungal keratitis from regions with a warm, humid climate and/or with an agricultural economy [5, 11]. This was evident by the present study in which, out of 69 patients with fungal keratitis, trauma was a predisposing risk factor in 54 (78.3) patients. Furthermore, of 76 fungal isolates, 61 (80.3 %) isolates were recovered from patients with trauma in which 68.4 and 11.9 % was accounted by molds and yeasts respectively. A relatively high prevalence of yeasts in trauma in the present study was not in agreement with earlier study that reported yeast infections typically occurs in eyes with preexisting ocular surface disease predominantly in temperate regions [28].

According to Sun et al. [28], the proportion of fungal keratitis caused by filamentous fungi increases towards tropical regions, whereas in many temperate regions, fungal keratitis appears to be rare and the most frequent etiologic agents are *Candida* species. This was evident by the present study in which, of the total number of fungal isolates mycelia fungi were the most common accounting for 82.9 % of the total isolates. Among mycelia fungi, *Fusarium* and *Aspergillus* species were the two predominant fungal isolates consisting of 27.6 and 25.0 % of the total isolates respectively, together comprising 52.6 % of the total isolates. This was followed by *Penicillium sp*, *Cladosporium sp and Scedosporium sp* consisting of 7.9 %, 6.6 and 5.3 % respectively. Similar findings of *Fusarium sp* as the leading filamentous fungal pathogen have been described in South Florida and Ghana [29], Southeast Brazil [27], North China [26] and Malaysia [30] where the climate is warm and humid like Ethiopia. This was, however, different from reports of *Aspergillus* sp from North India as was previously noted [31] and *Candida sp* from some developed countries [12, 32]. *Penicillium sp*, *Cladosporium sp and Scedosporium sp* were also reported as important cause of keratitis by similar studies [26, 27].

## Conclusion

Fungal keratitis was found out to be high. Male patients at the middle age were more affected than female patients. Similarly, patients from rural areas were more affected than from urban areas. Trauma was the most common predisposing risk factor. The infection was more prevalent in farmers. Patients in age groups of 15–35 years were significantly affect by the infection. High prevalence of fungal keratitis, highlights the need for nationwide study on fungal keratitis and precise identification of the causative fungal agents and institution of appropriate treatment strategy.
